# A bacterial food source for effective laboratory cultivation of the freshwater nematode *Plectus sambesii*

**DOI:** 10.17912/micropub.biology.001857

**Published:** 2025-10-04

**Authors:** Luke T. Geiger, Joke Evenblij, Oliver Hobert

**Affiliations:** 1 Department of Biological Sciences, Columbia University, New York, New York, United States; 2 Howard Hughes Medical Institute

## Abstract

The successful establishment of a new satellite animal model system depends in no small part on the ability to effectively cultivate such animals at scale in the laboratory. We describe here the discovery of a bacterial food source, *Pseudomonas putida* AAC02 *,* that improves laboratory cultivation of the parthenogenetic freshwater nematode *Plectus sambesii,* characterized by an embryonic patterning program, morphological features and environmental adaptations notably distinct from other presently well-established nematode model systems. We also describe the application of a freezing protocol for long-term storage of *Plectus sambesii* and several other emerging model species.

**Figure 1. Plectus sambessii and its food source Pseudomonas putida:**
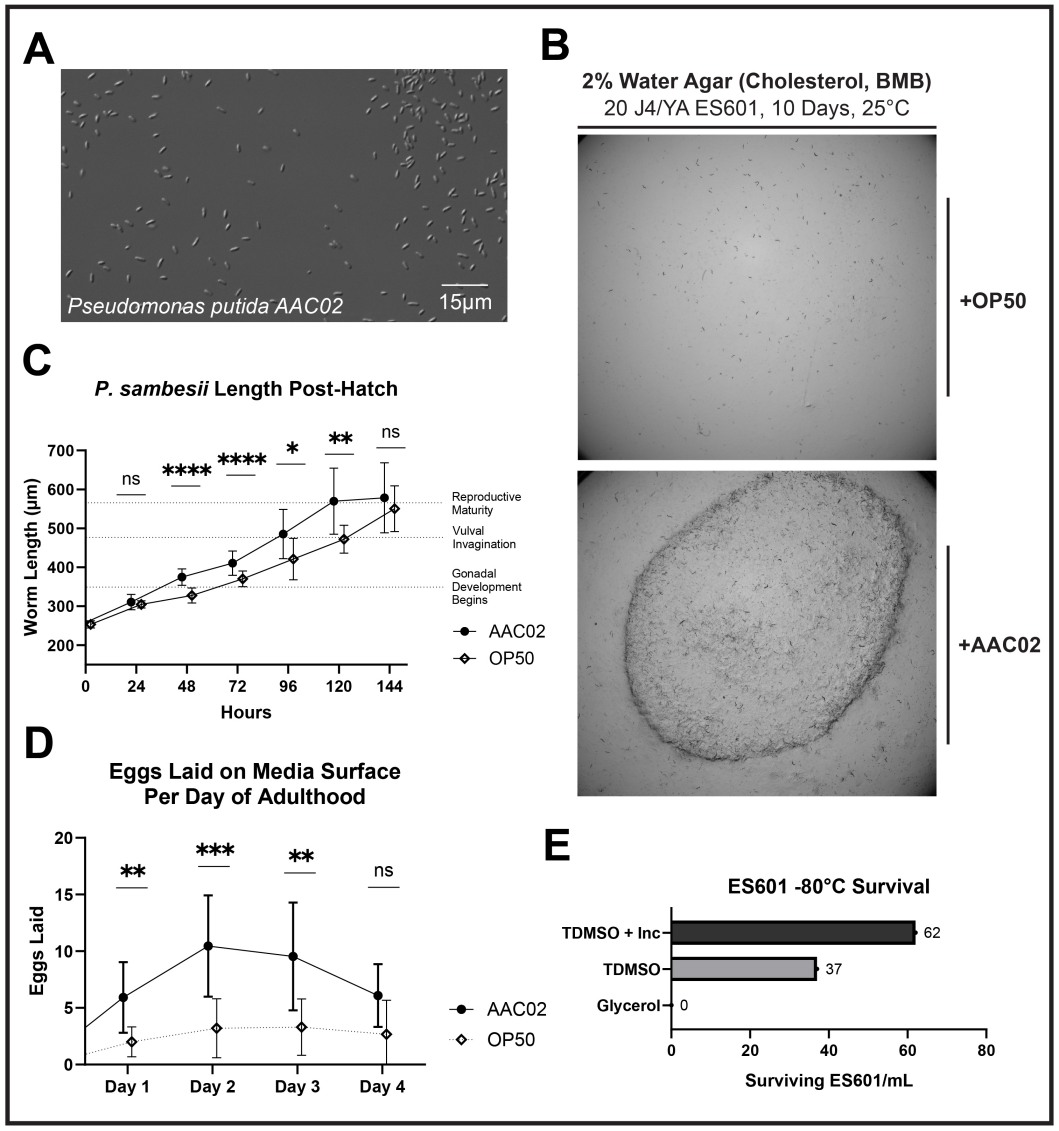
**A.** Nomarski contrast image of *Pseudomonas putida* AAC02. **B.** Representative image of cultures on water agar plates seeded with 100µL OP50 (top) or AAC02 (bottom). 20 J4/young adult ES601 animals were plated on either condition and left to grow for 10 days at 25°C. **C.** Body length in microns of ES601 cultured with either OP50 (n = 51, 28, 20, 15, 13, 12, 11 for observations at 0, 24, 48, 72, 96, 120, and 144 hours, respectively) or AAC02 (n = 49, 37, 37, 29, 23, 17, 21 for observations at 0, 24, 48, 72, 96, 120, and 144 hours, respectively). A mixed-effects model showed time of length measurement, food source, and the interaction term between time of length measurement and food source were significant (P < .0001, P < .0001, and P < .0001, respectively). **D.** Number of eggs laid per day by ES601 on low-salt agar seeded with either OP50 (n = 10) or AAC02 (n = 13). A mixed-effects model showed day of adulthood, food source, and the interaction term between day of adulthood and food source were significant (P < .0001, P < .0001, and P = .0053, respectively). **E.** Quantification of ES601 surviving either a glycerol-based freezing protocol, a trehalose/DMSO protocol, and the same trehalose/DMSO protocol with a 30-minute incubation step at room temperature. For all graphs, ns = not significant, * = P ≤ .05, ** = P ≤ .01, *** = P ≤ .001, **** = P ≤ .0001. Error bars indicate standard deviation. Observations on the same day are staggered for visual clarity in C and D.

## Description

Our laboratory aspires to establish the free-living freshwater nematode *Plectus sambesii* as a satellite model system. *Plectus sambesii* diverged from the classic nematode model system *Caenorhabditis elegans* more than 400 million years ago (Qing et al., 2024). This vast evolutionary distance is reflected in a peculiar pattern of embryogenesis, adaptation to a distinct ecological niche, divergent patterns of behavior, and the existence of various distinctive morphological characteristics compared to *C. elegans* (Schulze et a., 2012, Croll, 1971). These morphological characteristics include a distinct excretory system, the apparent absence of phasmids, and the presence of a spinneret opening and caudal gland cells, which allow *P. sambesii* to attach to substrates while underwater.

*Plectus sambesii* was originally isolated by Heinrich Micoletzky in the Zambezi River in Africa in 1916 (Micoletzky, 1916). More recently, an isolate from the Mekong River in Vietnam, ES601, has been cultivated under laboratory conditions using 2% water agar plates supplemented with cholesterol and seeded with OP50 bacteria (Lahl et al., 2002). We were able to corroborate that passaging animals using these culturing conditions can maintain a viable population of ES601 indefinitely. However, we found that the density of animals on plates was too low to readily isolate animals *en masse* for standard antibody staining or *in situ* mRNA staining protocols, or for cell or nuclei isolation protocols required for RNA sequencing. We therefore set out to find conditions under which we could culture ES601 at higher density.

While we observe ES601 can tolerate NGM for several days, our cultures maintained on NGM plates supplemented with OP50 tended to fail after one to two weeks. Instead, other researchers have cultivated the *Plectus sambesii* strain ES601 as well as other species in the genus *Plectus* on a number of different water agar preparations. These vary in the presence or absence of sand (Adhikari et al., 2010); trace salts and Bold's Modified Basal Freshwater Nutrient Solution (BMB) (Xu et al., 2022); a surface layer of water (Adhikari, 2009); and a microbial food source (de Tomasel et al., 2013, Kagoshima et al., 2012). We corroborate that many of these preparations were ultimately viable for the maintenance, but not the high-density culturing, of ES601.

We rationalized that importing features of a nematode's native environment into the laboratory would improve cultivation efficiency, as has recently been demonstrated for halophilic nematodes isolated from the Great Salt Lake in Utah (Michael Werner, personal communication). Since cyanobacteria are ubiquitous in the Mekong River delta from which ES601 was isolated (Nguyen et al., 2023), we assessed growth of ES601 on unseeded 2% water agar plates with cholesterol, BMB, and commercially obtained freshwater samples containing distinct cyanobacteria species (informally referred to as blue-green algae). We observed ES601 to grow and reproduce well on plates covered with freshwater containing the cyanobacterium *Anabaena sp.* Since we did not observe ES601 to consume the cyanobacteria, we hypothesized that the animals were consuming another microbe associated with the cyanobacteria culture. After streaking these freshwater samples with *Anabaena sp.* onto low salt agar plates with Bold's medium and ensuing multiple rounds of cloning out individual colonies, we isolated a bacterial strain from this commercial *Anabaena sp.* preparation which grows well on low-salt plates and is well-tolerated by ES601, which we designate AAC02 ( Fig. 1A ). We isolated DNA from this bacterial strain and subjected it to whole-genome sequencing to generate a high-coverage 6.2Mb genome assembly, which is deposited under NCBI BioProject ID PRJNA1313643 . Sequence comparison suggests AAC02 is a strain of *Pseudomonas putida* , with an overall similarity of 98% to *P. putida* strain INSali382. Alignment of annotated 16S rRNA gene sequences to other strains of *P. putida,* other *Pseudomonas* species, and *E. coli* supports this assignment (Ext. Data Fig. 1A- B). Whole genome sequencing for AAC02 also revealed copies of the *Yersinia pestis* plasmid pIP1203, which confers resistance to the antibiotic streptomycin. As natural antibiotic resistance is useful for both generating axenic cultures of AAC02 for seeding plates and limiting the contamination on plates during passaging, we incubated colonies of AAC02 overnight in LB at 37°C with and without streptomycin and confirmed the natural streptomycin resistance of our isolate (Ext. Data Fig. 1C ).

We further tested the relative performance of OP50 and AAC02 in culturing ES601 on our water agar plates. Visually, AAC02 forms a dense lawn which is optically amber but still allows for easy observation of ES601 on plates. In comparison, OP50 forms a very thin, clear lawn. We observed that the density of animals AAC02 can support on water agar plates is considerably higher than OP50 on the same plates ( Fig. 1B ). The higher density of AAC02 growth on low-salt agar plates compared to OP50 may also facilitate the mass-cultivation of other nematodes which prefer a similar media composition. While adult animals cultured on OP50 are eventually similar in size (~550-700 micrometers in length) to those cultured on AAC02, we sought out to quantify if culturing condition affects the time it takes worms to reach this size. Animals cultured with AAC02 grew to their terminal size in five days, for a total generation time of seven days ( Fig. 1C ). In contrast, animals reared on OP50 took six to seven days to reach adulthood after hatching, for a total generation time of about eight to nine days. The difference in length between AAC02 and OP50 was statistically significant at 48, 72, 96, and 120 hours (P < .0001, P < .0001, P = .0216, and P = .0021, respectively using a mixed-effects model and a Šidák multiple comparison post-hoc test). Worms were not significantly different in length both at the beginning and end of the measurement period, indicating both OP50 and AAC02 facilitate the complete growth of *P. sambesii* , though worms were longer earlier on AAC02. Additionally, the brood size of ES601 per day over the first four days of adulthood was 2.3-3.3x higher for worms reared on AAC02 compared to OP50 , and significantly higher for the first three days of adulthood (P = .0039, P = .0005, and P = .0034, respectively using a mixed-effects model and a Šidák multiple comparison post-hoc test) ( Fig. 1D ). The viability of individual eggs laid under both conditions appeared qualitatively comparable.

A key advantage of *C. elegans* as a model system is the ability to freeze and later recover viable animals. We tested whether *P. sambesii* could be frozen and successfully recovered after thawing using a standard *C. elegans* freezing protocol with glycerol. While no animals were recovered following this freezing protocol, adopting a modified trehalose-based protocol (Methods – *Freezing Protocol* ) outlined in (O'Connell et al., 2022) improved the viability of frozen animals dramatically ( Fig. 1E ). We also notice that this modified trehalose freezing protocol generally improves the recovery of other satellite models, including *Pristionchus pacificus* and *Steinernema hermaphroditum* .

## Methods

*Water Agar Plate Preparation*

1. Add 20mL of 1x Bold's Modified Basal Freshwater Medium to 20g of agar and fill to 1L with ddH20.

2. Autoclave mixture on a 30-minute liquid cycle.

3. Add 1mL of cholesterol (poured from a stock of .5g in 100mL 96% EtOH) when mixture temperature is lukewarm, as well as 1.25 mL nystatin in 70% ethanol.

4. Pour the solution into petri dishes in a laminar hood, about 10mL per 6cm plate.

5. Let plates sit for 2-3 days at room temperature to harden and release moisture before seeding. Plates can be stored at 4°C for several months prior to use.

*Inoculation of Water Agar Plates with AAC02*

1. Pick a single colony of AAC02 from a streaked plate into 500mL autoclaved LB solution.

2. Incubate colonized LB solution overnight at 37°C.

3. Transfer a working stock of bacteria from the incubated LB solution into a sterile 50mL centrifuge tube and retain the remainder at 4°C for up to 2-3 months.

4. Seed water agar plates with up to 400µL of AAC02. Incubate seeded plates overnight at 37°C to facilitate initial lawn growth.

5. Remove seeded plates from incubator and allow 2-3 days at the benchtop for further lawn development before use with nematodes.

*Isolation and Identification of AAC02 from a Commercial Culture of* Anabaena sp.

AAC02 was isolated by streaking water from a commercial preparation of *Anabaena sp.* (maintained in Alga-Gro® Freshwater) on 2% water agar plates supplemented with cholesterol and BMB, then incubating them overnight at 37°C. The thickest single colonies were picked and incubated individually in LB overnight at 37°C. Cultures grown in LB were subject to the same singling procedure two additional times in order to isolate an axenic culture of a microbe which was amenable to laboratory cultivation and growth on media preferred by ES601. Following a fourth and final streaking and growth in LB, the most promising isolate was sent for commercial sequencing and analysis. Bacterial genome sequencing was performed by Plasmidsaurus using Oxford Nanopore Technology with custom analysis and annotation, which suggested *Pseudomonas putida* as the best match for our isolate. All seven gene homologs for sequences annotated as “16S Ribosomal RNA” by this pipeline in the AAC02 genome assembly were queried using a BLAST megablast against “refseq_rna”, which returned almost exclusively hits for *Pseudomonas* species. Broadening the BLAST megablast search to “nt” returned almost exclusively best matches for *Pseudomonas putida* strains, though no strain entirely matched our isolate.

The multiple sequence alignment of AAC02, *P. laurentiana* GSL-010 (NR_179898.1), *P. aeruginosa* PA01 (DQ777865.1), *P. putida* N1R (LT707061.1:2429312-2430848), and *E. coli* (J01859.1) 16S ribosomal RNA gene sequences in Extended Data Figure 1A- B was performed with Clustal Omega.

Streaked plates of AAC02 are available from our laboratory upon request.

*Brood Size Assay*

J4 animals were singled onto plates seeded with either OP50 or AAC02, then replated every 24 hours. Eggs laid on the surface of each plate were counted after replating. Only worms which were recovered from the top surface of the agar and successfully replated for all four days of adult observation were included in the final quantification and analysis. The generation time of *P. sambesii* means that worms picked during early J4 might not be reproductively mature 24 hours later for the first observation period. Results from these animals were shifted back 24 hours to create a “day zero” and guarantee that all subsequent measurements corresponded to the same day of adulthood for all worms. The drop out rate of individual animals across the experiment (approximately 75% by the fourth day of adulthood) made observation past day four technically challenging, though individual adults continue to lay eggs for at least a week.

*Obtaining a Synchronized Population of ES601 + Growth Assay*

Plates with ES601 underwent a standard egg prep (Stiernagle, 2006), except bleach solution exposure was limited to 2 minutes in order to only kill adult worms but not significantly affect the eggs laid on washed plates. This modification was made to improve arrested J2 yield because adult ES601 are not particularly gravid. Eggs were allowed to hatch over 48 hours at room temperature (approx. 21-23°C) in a 5% M9/ddH2O solution. Ten synchronized J2 animals were deposited on plates seeded with 100 µL of either OP50 or AAC02 and imaged every 24 hours on a Nikon Eclipse E400 at 20x magnification. Measurements of body length were taken from these images with Fiji 1.54p using the segmented line tool to closely follow the centerline of each animal from mouth to spinneret opening, then converting pixel distances to a measurement standard. Not all animals were visible for imaging at each timepoint.

*Freezing Protocol*

Two plates with many well-fed young (J2) animals were washed with M9 and divided evenly into three groups for freezing. Glycerol freezing was performed as described in Stiernagle 2006. The trehalose-DMSO freezing protocol was conducted essentially as described in (O'Connell et al., 2022), including footnotes regarding a room temperature incubation.

## Reagents

Bold's Modified Basal Freshwater Nutrient Solution, 50x, 500mL, B5282, Sigma-Aldrich

Cyanobacteria Set, Item #: 151515, Carolina Biological Supply

## Data Availability

Description: Extened Data Figure. Resource Type: Image. DOI: https://doi.org/10.22002/fcdty-shv13
